# A moonshot for diabetes and obesity

**DOI:** 10.1016/j.igie.2025.09.013

**Published:** 2025-10-03

**Authors:** Linda S. Lee, Jay Caplan, Harith Rajagopalan

**Affiliations:** 1 Division of Gastroenterology, Hepatology and Endoscopy, Brigham and Women's Hospital, Boston, Massachusetts, USA; 2 Fractyl Health, Burlington, Massachusetts, USA

## Editor's introduction

Currently, over 820 million adults have diabetes mellitus worldwide, which is an over 4-fold increase since 1990.^1^ In parallel, more than 1 billion people worldwide are living with obesity, a condition that significantly increases the risk for developing type 2 diabetes mellitus (T2D) and other metabolic diseases. Together, these twin epidemics represent one of the most urgent global health challenges of our time.^2^ Over 90% of patients with diabetes have T2D. Remarkably, nearly 60% of adults with diabetes mellitus are untreated, with 90% of these untreated patients living in low- to middle-income countries.^3^ Similarly, only 1.6% of obese people in the United States reported having any form of treatment.^4^ Management of T2D focuses on lifestyle modification, medications, and prevention of adverse events. Although glucagon-like peptide-1 (GLP-1) agonists have seemingly revolutionized management of T2D and obesity, limitations include adverse effects and the need to remain on the medication long term. The increased morbidity and mortality associated with diabetes and obesity are well documented.

Dr Harith Rajagopalan and Jay Caplan co-founded Fractyl Health (Burlington, Mass, USA) with a vision to address the root causes of T2D and obesity by leveraging endoscopic approaches. Dr Rajagopalan is CEO of Fractyl Health and started the company while serving as an Entrepreneur-in-Residence at General Catalyst Partners (Cambridge, Mass). Previously, he was an academic cardiologist and physician-scientist. He received his BS in chemistry from Stanford University and went on to obtain MD and PhD degrees from the Johns Hopkins School of Medicine. After medical school, Dr Rajagopalan trained in internal medicine and clinical cardiology at Brigham and Women's Hospital and completed a research fellowship at the Harvard Stem Cell Institute, Harvard Medical School. Dr Rajagopalan and his family live in Wellesley, Massachusetts.

Jay Caplan is President and Chief Product Officer of Fractyl Health and has held leadership positions in the medical device field for over 20 years. Previously, he was the Chief Operating Officer of Candela Corporation (Marlborough, Mass), a world leader in esthetic devices. He also served as Chief Technology Officer and Vice President of Research and Development at Infraredx, Inc (Bedford, Mass), a venture-backed start-up developing a novel cardiovascular imaging system to identify lipid-rich coronary plaques. Before Infraredx, Jay was Vice President of Operations at Thermo Cardiosystems (Woburn, Mass), where he led the team developing the HeartMate II left ventricular assist device. Jay holds a BS in electrical engineering from the Massachusetts Institute of Technology and an MBA from The Wharton School of the University of Pennsylvania.

**Figure 1:**
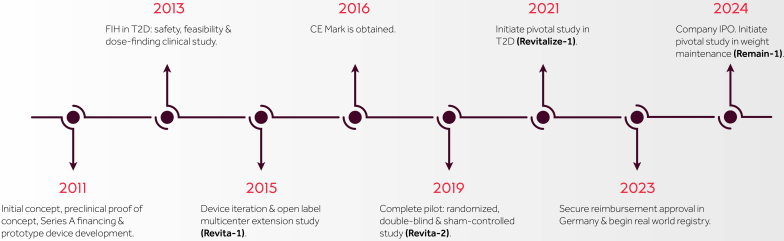
Timeline of key Fractyl (Burlington, Mass, USA) events. *CE*, Conformité Européenne; *FIH*, first-in-human; *IPO*, initial public offering; *T2D*, type 2 diabetes mellitus.

**Linda S. Lee (LL):** Would you share how Fractyl came about and how the name “Fractyl” was selected (Fig. 1)?

**Harith Rajagopalan (HR):** Fractyl Health was founded with a singular goal: to leverage therapeutic endoscopy to address the root causes of metabolic disease, specifically obesity and T2D. Inspired by the durable metabolic improvements seen after bariatric surgery, we asked a transformative question: Could a less-invasive, endoscopic procedure achieve similar effects by targeting the duodenal mucosa, a key site of metabolic dysfunction?

The name “Fractyl” reflects the company's ethos of innovation and complexity. Inspired by the concept of fractals—mathematical structures that reveal intricate, repeating patterns regardless of scale—our name symbolizes the profound complexity of the human body and the elegant simplicity of our approach to addressing it. The intestinal mucosa is a fractal, with plicae circulares leading to villi and microvilli at higher levels of magnification. The pancreatic islet is a fractal, with islets of Langerhans leading to individual spherical cells containing vesicles of nutrient-stimulated hormones within them. We are inspired by the fractals in natural phenomena as we seek to decode and intervene in the fundamental biological processes driving metabolic disease. The name also conveys our commitment to breaking free (“fracture”) from traditional patterns of chronic disease management, pioneering a new era of transformative health care solutions. This vision is deeply aligned with the gastrointestinal (GI) advanced endoscopy community, whose expertise and innovative spirit will be pivotal in realizing the potential of our solutions.

**LL:** What were the early days like? Where were you meeting, doing your experiments?

**HR:** Fractyl began humbly. We had a 200-square-foot office in a 100-year-old watch factory with a great view but terrible ventilation. We had a refrigerator that was supposed to be used for our lunches but sometimes housed materials for our preclinical experiments (I may get in trouble if I reveal more than that). When we finally moved into our first real space, we all assembled our own Ikea desks and used our own credit cards to order our computer equipment.

Our first major engineering hurdle was to design a catheter that could deliver uniform and safe mucosal ablation while preserving submucosal integrity. The duodenum, although central to metabolic regulation, posed significant anatomical and technical challenges for us to navigate: it is anatomically variable, has a high risk of adjacent organ involvement, and presents limited maneuverability with standard endoscopic equipment. Early prototype testing in rodent models at a local contract research organization revealed promising metabolic improvements, encouraging us to pursue larger animal studies and ultimately our first-in-human (FIH) clinical trial in 2013. The positive findings from this feasibility study laid the groundwork for future device iterations and multicenter clinical studies.

**Figure 2:**
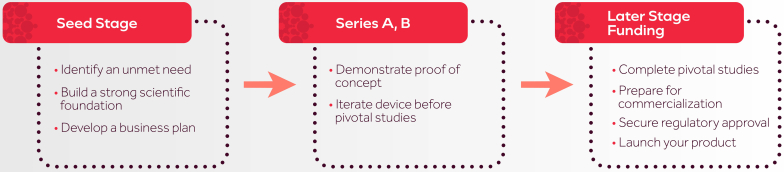
How funding works and the different steps.

**LL:** Funding is certainly a critical part of all start-ups. Would you discuss how funding has worked for Fractyl as well as the various steps in the process of securing funding and the timing and thinking behind your recent initial public offering (Fig. 2)?

**HR:** Funding at Fractyl has always been aligned with our key milestones. Initial seed and Series A investments supported early prototyping, preclinical studies,^5^ and our first FIH clinical trial.^6^ Series B and C funding enabled us to scale up operations, refine the Revita device through design iterations, and conduct the REVITA-2 study—a double-blind, sham-controlled trial that validated the safety and efficacy of duodenal mucosal resurfacing.^7^

Most recently, our 2024 initial public offering allowed us to broaden our investor base and secure the capital needed to launch our pivotal study in weight maintenance following GLP-1 discontinuation. Although our earlier clinical programs focused on type 2 diabetes, the insights gained from those studies have directly informed this next phase of development, which aims to sustain metabolic benefits after GLP-1 discontinuation. This latter study builds on a growing body of evidence demonstrating that endoscopic modulation of the duodenal mucosa offers a durable, nonpharmacologic approach to metabolic disease.

**LL:** A well-functioning, cohesive team is important especially in smaller start-ups. How have you developed your team?

**HR:** Building a cohesive and high-performing team has been one of the most critical and rewarding aspects of Fractyl's journey. From the outset, we have hired individuals who not only bring expertise in their fields but also share a deep sense of mission and commitment to transforming the lives of patients with obesity and T2D. Fractyl's team reflects a fusion of procedural, scientific, and technical expertise. We have recruited GI endoscopists, device engineers, clinical trialists, and gene therapy scientists to collaborate closely in shaping our products into procedures that are intuitive, reproducible, and impactful.

Our culture is one of first principal thinking, humanistic perspective toward patients living with chronic disease, audacity to challenge the status quo, and tenacity to deliver real solutions to patients.

**LL:** What have been the biggest challenges to date?

**HR:** Introducing novel, procedure-based therapies for metabolic disease required a shift in both clinical thinking and technical execution. Early on, the notion that the duodenum could drive systemic insulin resistance was met with skepticism. We addressed this by investing in mechanistic research that demonstrated how modern diets remodel the duodenal mucosa, altering nutrient sensing and hormone signaling in ways that promote metabolic disease.

**Figure 3:**
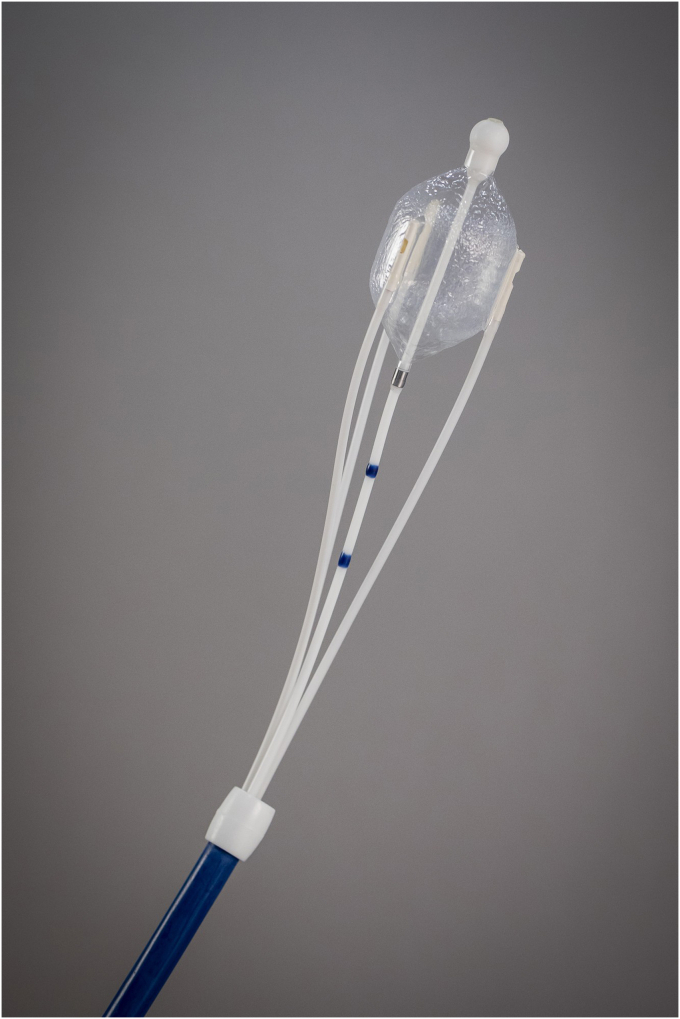
Original dual catheter for duodenal ablation.

**Figure 4:**
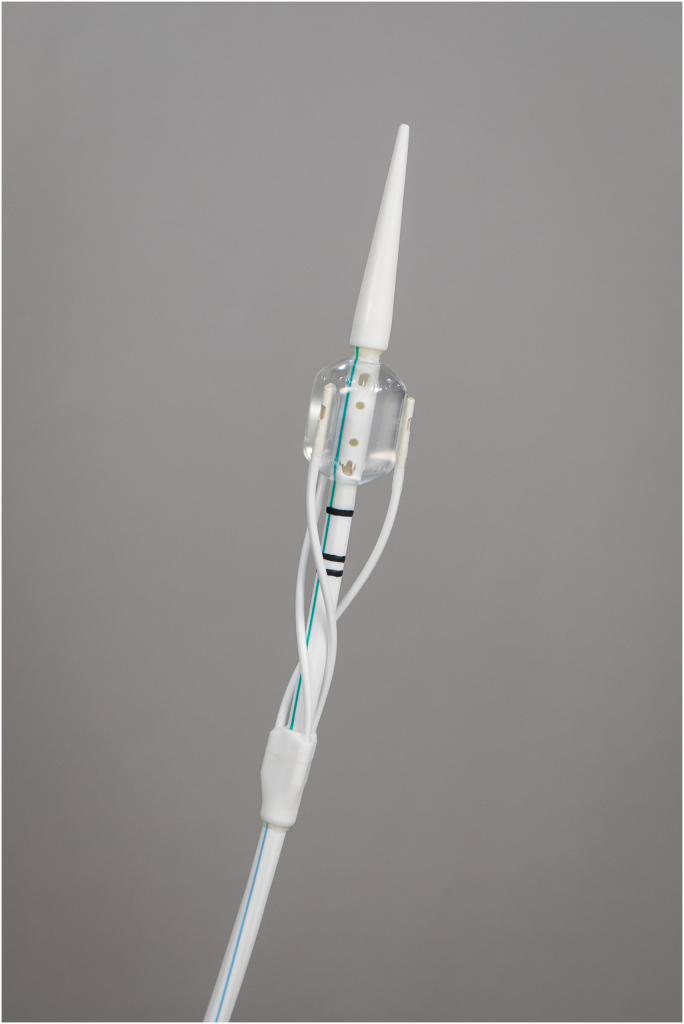
Simplified single catheter for duodenal ablation.

**Figure 5:**
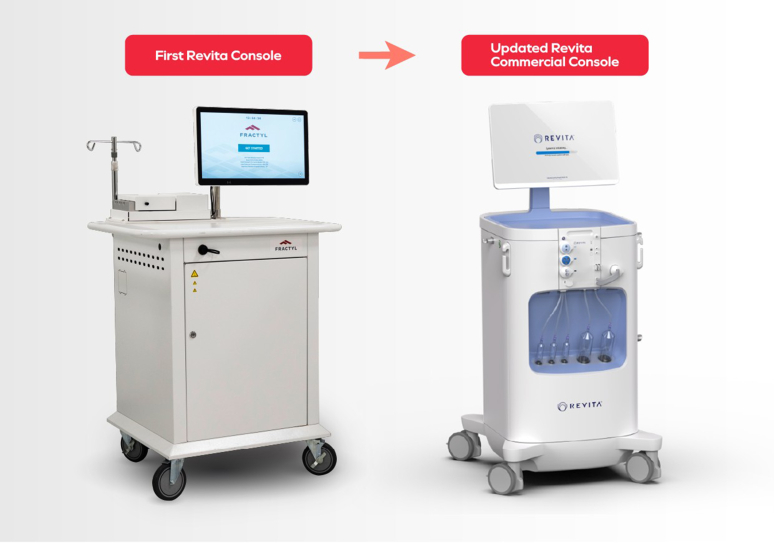
First Revita console and updated commercial console (Fractyl, Burlington, Mass, USA).

Procedurally, designing a reliable ablation system for the duodenum demanded several generations of catheter prototypes—evolving from dual-catheter configurations (Fig. 3) to a simplified, single-catheter system (Fig. 4), and now we are very excited about an upcoming new development, which is a through-the-scope duodenal ablation catheter. We also developed a custom clinical console to support safe and standardized energy delivery (Fig. 5). Feedback from endoscopists has been instrumental in optimizing catheter handling, visualization, and safety features. Human factor testing, bench simulations, and early clinical data have shaped a training program to ensure consistent results across a variety of clinical settings.

Adoption of Revita within the GI community will require more than just strong data; it requires workflow integration. We have been working closely with physicians to ensure that the procedure could be performed with standard endoscopy equipment and within typical outpatient settings.

Other major challenges we faced along the way were totally unpredicted but led to unexpected new opportunities. The impact of coronavirus disease 2019 on shutting down endoscopy centers completely halted our clinical trial progress, but enabled us to start working on a new and exciting pancreatic gene therapy platform called Rejuva. The explosion of GLP-1s in obesity affected our T2D clinical studies but opened the door to a post-GLP-1 weight maintenance therapeutic option. In the end, these challenges created whole new avenues we could never have otherwise imagined.

**LL:** Harith, you are a physician by training and changed courses. When did you realize that entrepreneurship was your passion? For those interested in a similar path, what is your advice on how to get started?

**HR:** As a physician-scientist, I always imagined my path would lead me to academic medicine, focusing on research and patient care in a traditional setting. But over time, I became increasingly moved by the vision that we could offer something profoundly better for patients by addressing the root causes of disease rather than just managing symptoms. This idea—that we could fundamentally rethink how we treat complex diseases like obesity and T2D—captivated me and ultimately propelled me into entrepreneurship. I realized that the tools and approaches we were using in medicine were simply not enough to break the pattern of these chronic conditions, and that innovation outside the conventional pathways was needed to make a real impact.

For those considering a similar path, my strongest advice is to pursue entrepreneurship not as an escape from medicine, but as an affirmative desire to make a different kind of impact in the world. Entrepreneurship is not easy—it requires resilience, creativity, and a willingness to take risks—but if you are driven by a mission that you deeply believe in, it can be incredibly rewarding. Start by identifying the problem you are passionate about solving, immerse yourself in understanding it from every angle, and then build the skills and relationships necessary to create a solution. The most meaningful relationships come from authentic collaboration with people who share your commitment to solving hard problems—seek out mentors and peers who challenge and push you. On skills, do not be afraid to step outside your comfort zone; I had to learn business, regulation, and finance on the fly, and every misstep was a chance to grow. If you stay curious, humble, and mission-driven, the ecosystem you need will form around you. Most importantly, stay grounded in your purpose and let the desire to make a difference guide your journey.

**LL:** Jay, how does an electrical engineer end up working on obesity and diabetes? For those not in medicine who wish to pursue this type of work in medicine, what is your advice?

**Jay Caplan (JC):** As an engineer and MBA by training, I have always been drawn to solving complex, real-world problems through innovative technology. Harith and I met through Fractyl's earliest seed-stage investors, venture capitalists whom we had both gotten a chance to meet separately. The venture capital partner felt that we could bring our complementary experiences (mine in engineering and business, and Harith's in science and medicine) to co-found a transformative new company. When Harith and I began discussing the growing epidemic of obesity and T2D, it became clear that these conditions were not being addressed at their root cause—and that there was a unique opportunity to apply systems thinking to an area of medicine in desperate need of new solutions. The transition from engineering to health care was not as far of a leap as it might seem. At its core, engineering is about problem-solving, and this was one of the most compelling challenges I had encountered: how to design technologies that could fundamentally change the course of metabolic disease.

My background in electrical engineering gave me a solid foundation in understanding systems and designing solutions that are both efficient and reliable. This perspective became critical in developing the Revita system, where precision, safety, and usability are paramount. My time at Wharton further equipped me to navigate the complexities of building a business, including securing funding, managing growth, and working with diverse stakeholders. At Fractyl, it has been incredibly rewarding to bring together these skills in engineering and business to create products that have the potential to profoundly impact patients' lives.

For those not in medicine who are considering a similar path, my advice is to focus on where your skills and passions align with an unmet need in health care. You do not need a medical degree to make a difference in medicine, but you do need a deep understanding of the problems you are trying to solve. Surround yourself with experts in the field—clinicians, researchers, and patients—and immerse yourself in their perspectives. At the same time, do not be afraid to bring your unique expertise to the table. Health care desperately needs fresh thinking from diverse disciplines, and engineers, entrepreneurs, and technologists have a vital role to play in driving innovation. The key is to approach the work with humility, curiosity, and a commitment to making a meaningful impact.

**LL:** There seems to be a lot of uncertainty in the world of start-ups. How do you manage this and try to minimize the risks?

**HR:** Uncertainty in health care innovation is inevitable. Our strategy has been to de-risk our approach through systematic progress: mechanistic validation, iterative prototyping, and controlled clinical studies. Regulatory milestones such as U.S. Food and Drug Administration Breakthrough Device designation and Conformité Européenne mark approval reflect external validation of this risk-reduction strategy along the journey.

Although we cannot eliminate all uncertainty, we focus on controlling what we can: rigorous science, thoughtful design, robust clinical evidence, and strong relationships and dialogue with regulators and clinicians. By breaking the journey into smaller, achievable steps, we minimize risks and build a solid foundation for the future.

**LL:** What are the next steps for Fractyl?

**HR:** Fractyl has completed enrollment in its pivotal U.S. study for Revita in post-GLP-1 weight maintenance. These data will be foundational to U.S. Food and Drug Administration submission and broader clinical adoption.

In parallel, we are developing Rejuva, our next-generation gene therapy platform, with a “smart GLP-1” with the potential for durable remission of T2D and obesity. The idea behind Rejuva is to locally deliver low doses of gene therapy directly to the pancreas to allow genetic modification of the pancreas for the first time, to our knowledge. Our first candidate in the Rejuva platform, RJVA-001, is a “smart GLP-1,” a nutrient responsive version of the human GLP-1 hormone that can be produced in the pancreatic beta cell and secreted whenever insulin is secreted. You can think of it as using gene therapy to turn the beta cell into a gut L cell to increase endogenous GLP-1 expression and secretion. Preclinical studies have shown very impressive and durable improvements in body weight and blood sugar, even when compared with semaglutide, and the results open the door to a 1-and-done GLP-1 treatment for metabolic disease. RJVA-001 is expected to enter the clinic in 2026 for an FIH study in T2D. If successful, we believe that Rejuva represents a transformative potential future direction for endoscopically delivered biologics with profound efficacy.

We believe that GI endoscopists are uniquely positioned to lead a new era in metabolic care. With the right tools and evidence, endoscopy can evolve from a diagnostic modality to a therapeutic platform that transforms chronic disease management.

## Editor's closing remarks

Traditionally, when we consider endoscopic therapy, we focus on the immediate intestinal, pancreatobiliary, vascular lesion that we can directly visualize and treat. Bariatric endoscopy, where endoscopic sleeve gastroplasty has led to improvements in diabetes and obesity, has certainly brought to the forefront the concept of endoscopic therapy impacting non-GI tract diseases.^8^ Although exciting, endoscopic sleeve gastroplasty remains a skill and time-intensive procedure with limited availability. The idea of having a simple endoscopic procedure that many gastroenterologists and/or advanced endoscopists can perform to impact T2D and obesity is certainly exciting and will hopefully become a reality.

## Disclosure

The following authors disclosed financial relationships: H. Rajagopalan and J. Caplan: Employee and shareholder of Fractyl Health. L. S. Lee: Consultant for Boston Scientific, Fujifilm Healthcare Americas, Cook Medical, and Micro-Tech Endoscopy; consultant and research grant recipient for Medtronic.
